# EEG vs. Hybrid EEG–fNIRS BCI for FES Control in Healthy Subjects: A Blind Randomized Study and an Open Dataset

**DOI:** 10.3390/s26175617

**Published:** 2026-09-04

**Authors:** Olesya Mokienko, Evgeniy Lukyanov, Leonid Kim, Mikhail Isaev, Gregory Gabuzov, Dmitry Bobrov, Roman Lyukmanov, Natalia Suponeva, Ksenia Ustinova, Pavel Bobrov

**Affiliations:** 1 Department of Neuro-computer Interfaces, Pirogov Russian National Research Medical University, Moscow 117513, Russia; mokienko_oa@rsmu.ru (O.M.); lukianov_es@rsmu.ru (E.L.); isaev_mr@rsmu.ru (M.I.); gabuzov_gg@rsmu.ru (G.G.); bobrov_da@rsmu.ru (D.B.); bobrov_pd@rsmu.ru (P.B.); 2 Institute for Neurorehabilitation and Restorative Technologies, Russian Center of Neurology and Neurosciences, Moscow 125310, Russia; xarisovich@gmail.com (R.L.); suponeva@neurology.ru (N.S.); 3 Institute of Biomedical Engineering, University of Science and Technology “MISIS”, Moscow 119049, Russia; yfvyfv@mail.ru; 4 Department of Physical Therapy, Central Michigan University, Mount Pleasant, MI 48859, USA

**Keywords:** brain–computer interface, near-infrared spectroscopy, electroencephalography, electric stimulation

## Abstract

**Highlights:**

**What are the main findings?**

- Using a real-time classical decoding pipeline, hybrid EEG–fNIRS BCI did not outperform EEG-only BCI for FES control.
- EEG C3/C4 were the most informative channels, while fNIRS placement required individual optimization.

**What are the implications of the main findings?**

- EEG-only BCI may be sufficient for practical BCI–FES use.
- The open dataset can support future BCI algorithm development and benchmarking.

**Abstract:**

Among the various brain–computer interface (BCI) modifications used in post-stroke rehabilitation, BCI systems combined with functional electrical stimulation (FES) are considered the most effective. As a preliminary step toward optimizing such systems for clinical application, it remains unclear whether using electroencephalography (EEG) alone versus a hybrid EEG and functional near-infrared spectroscopy (fNIRS) approach affects real-time three-class BCI–FES control performance in healthy individuals. In a blind randomized study, 16 healthy volunteers completed five BCI–FES training sessions across three days. In one group, FES of wrist extensor muscles was driven by a hybrid EEG–fNIRS classifier; in the other, by EEG only. Classification accuracy, sense of agency, attention, and physical comfort were assessed. No statistically significant between-group differences were found in any outcome measure (*p* > 0.05). Median real-time three-class classification recall was 53.5% in the hybrid group and 57.3% in the EEG-only group. The median agency score reached approximately 75% of the maximum possible value in both groups. Simulation analysis showed comparable accuracy for unimodal fNIRS-only and EEG-only classifiers. Genetic algorithm-based channel selection identified C3 and C4 as the most informative EEG channels, while optimal fNIRS placement required individual optimization. Within the constraints of the classification and fusion pipeline used here, these findings suggest that signal acquisition modality does not significantly influence BCI–FES performance or sense of agency in healthy subjects. The complete EEG–fNIRS dataset is publicly available through NITRC.

## 1. Introduction

A brain–computer interface (BCI) is a system that measures brain activity and converts it in (nearly) real-time into functionally useful outputs to replace, restore, enhance, supplement, and/or improve the natural outputs of the brain [1]. In post-stroke rehabilitation, BCIs enable patients to receive contingent feedback during motor imagery or movement intention, driving activity-dependent neuroplasticity and supporting motor recovery [2]. Functional electrical stimulation (FES) is considered the most physiologically congruent feedback modality, as it synchronizes cortical motor activation with actual contraction of paretic limb muscles [3]. This temporal coupling is thought to engage Hebbian learning mechanisms that may underlie the therapeutic effects of BCI–FES [4]. This interpretation is supported by clinical evidence: several meta-analyses of randomized controlled trials confirm that BCI–FES achieves the largest effect sizes for upper limb functional recovery compared to BCI systems employing robotic or visual feedback [5,6,7].

Brain activity in non-invasive BCIs can be recorded using several modalities. The most widely adopted is electroencephalography (EEG), which typically captures event-related desynchronization (ERD) of the mu rhythm over the contralateral sensorimotor cortex, providing a millisecond-resolution index of cortical engagement during motor imagery. Functional near-infrared spectroscopy (fNIRS) measures hemodynamic correlates of neural activity: motor imagery evokes a characteristic increase in oxyhemoglobin and a decrease in deoxyhemoglobin concentration in corresponding cortical regions [8,9]. Compared to EEG, fNIRS is less susceptible to electrical interference and motion artifacts; however, its temporal resolution is inherently limited by the sluggishness of the hemodynamic response [10]. These contrasting profiles have prompted the development of hybrid EEG–fNIRS BCIs, which aim to offset the limitations of each individual modality and improve the accuracy of motor imagery decoding [11]. Despite growing interest, the practical advantages of hybrid EEG–fNIRS configurations over conventional EEG-only BCIs for triggering FES remain insufficiently evaluated [11], and the optimal number of EEG and fNIRS channels for three-class motor imagery decoding has not yet been established. Even in studies conducted exclusively in healthy volunteers, evidence supporting the superiority of hybrid EEG–fNIRS BCIs remains limited by important methodological constraints. Most EEG–fNIRS BCI studies have relied on offline signal processing and classification [12,13,14,15,16,17,18,19,20], which is known to overestimate real-world decoding accuracy relative to genuine real-time, closed-loop implementations. Furthermore, the few studies that have implemented online classification have typically been restricted to binary classification tasks distinguishing only two states [21,22,23,24]. To our knowledge, no prior study has directly compared a hybrid EEG–fNIRS BCI against an EEG-only BCI under online, three-class, closed-loop conditions specifically for FES control.

The aim of this study was to compare objective and subjective performance metrics of EEG-only and hybrid EEG–fNIRS BCIs, operating within a three-class paradigm, for real-time control of hand muscle electrical stimulation in healthy subjects. Using a simulated fNIRS feedback approach, we also modeled and evaluated fNIRS-only BCI decoding accuracy and compared it with EEG-only BCI performance. We additionally aimed to identify the optimal number and placement of EEG and fNIRS channels for three-class motor imagery decoding in a BCI–FES paradigm. Based on findings in healthy subjects, we propose recommendations for the BCI–FES configuration most suitable for subsequent clinical trials. Finally, we present an open dataset of EEG and hybrid EEG–fNIRS recordings acquired during BCI–FES control sessions in this study.

## 2. Materials and Methods

### 2.1. Study Design

This study was conducted as a blind, prospective, randomized controlled trial designed to compare the control accuracy of an EEG-based BCI with that of a hybrid EEG–fNIRS BCI for triggering electrical stimulation of hand muscles in healthy volunteers, with each hand stimulated separately in alternating trials. The study protocol was approved by the Local Ethics Committee of Pirogov Russian National Research Medical University (extract No. 255, 17 November 2025).

### 2.2. Subject Eligibility Criteria

Healthy participants were recruited according to the following criteria.

Inclusion criteria:

- Voluntary written informed consent to participate in the study;
- Male or female sex;
- Age between 18 and 65 years;
- No acute illness and no exacerbation or relapse of chronic diseases at the time of inclusion;
- Score > 0 on the Edinburgh Handedness Inventory.

Non-inclusion criteria:

- Refusal to participate in the study;
- Use of medications affecting the nervous system at the time of the study (with the exception of antidepressants and anxiolytics with a stable dose and no initiation of a new drug within the last 30 days);
- Presence of a cardiac pacemaker;
- Presence of metallic implants or any other non-removable metal (piercings, superficial surgical clips) in the upper limb area;
- Skin diseases or injuries in the area of the scalp or upper limbs;
- Edema or signs of vascular disease of the upper limbs (redness, cyanosis, varicose veins).

Exclusion criteria:

- Voluntary withdrawal from the study;
- Development of an acute illness or exacerbation of a chronic disease;
- Initiation of new, or change in dosage of previously taken, medications acting on the central nervous system;
- Absence of a visible motor response to electrical stimulation;
- Intolerance to cutaneous electrical stimulation.

### 2.3. BCI System

**Figure 1:**
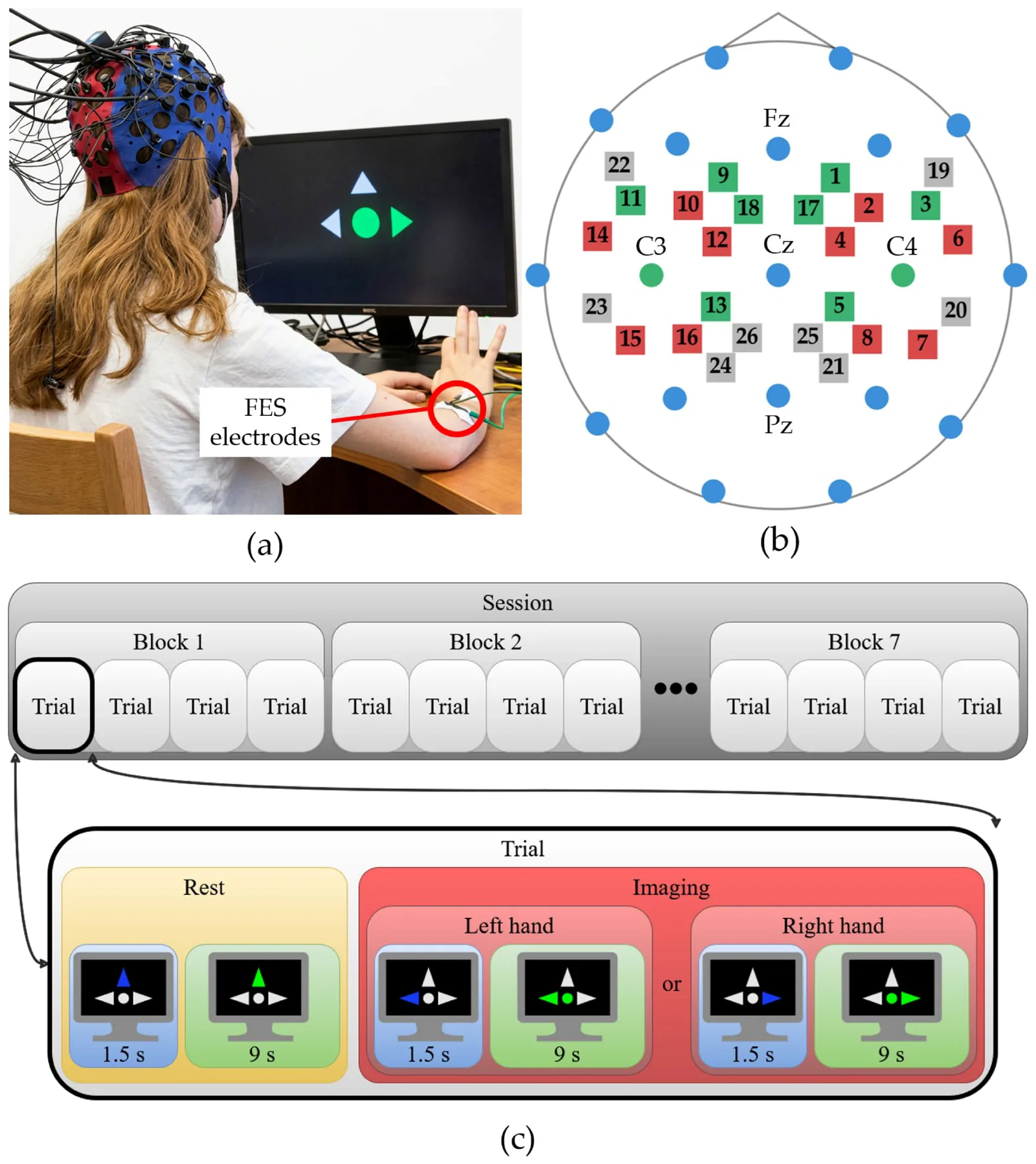
System setup (**a**). Sensor placement (**b**): circles indicate EEG electrode positions; squares indicate fNIRS channel positions. Red and green fNIRS channels were present in all sessions, while gray channels were partially absent. Green EEG and fNIRS channels represent the reduced subset selected via genetic algorithm search for the most informative channels, used in the classification simulation. Session structure (**c**). Blue arrows indicate the preparation and green arrows indicate the active task.

The BCI system consisted of a laptop with an additional monitor for cue presentation, EEG and fNIRS acquisition devices, and an electrical stimulator to provide FES feedback. Cue presentation, data acquisition, processing, and classification pipelines were implemented in MATLAB 2022b (Mathworks Inc., Natick, Massachusetts, USA). Temporal alignment of the EEG and fNIRS data streams was achieved using the Lab Streaming Layer (LSL) framework. The system setup is shown in Figure 1a.

### 2.4. BCI-Controlled Electrical Stimulation Procedures

Each participant completed three BCI–FES visits on separate days, each lasting 50–60 min including electrode setup. The first visit comprised one session, while the second and third each comprised two sessions, for a total of five training sessions.

In the first group, BCI-driven electrical stimulation was controlled exclusively using EEG-derived brain activity signals. In the second group, both EEG and fNIRS signals were used to drive FES. To maintain blinding, all participants wore identical caps with the same number of EEG electrodes and fNIRS optodes regardless of group assignment. In the EEG-only group, fNIRS data were recorded but excluded from the control pipeline. In the hybrid group, both modalities contributed to triggering FES. The experimenter administering the sessions was blinded to group allocation. Blinding was implemented by assigning each participant a randomly drawn code (“Heads” or “Tails”), which determined which software configuration was launched without revealing the condition to the experimenter.

Cortical activity was recorded using a 21-channel wireless EEG system (NeoRec 21 mini, Medical Computer Systems LLC, Zelenograd, Moscow, Russia) combined with an fNIRS system comprising 16 sources and 8 detectors (NIRx Medical Technologies, Orlando, Florida, USA). Conductive gel was applied beneath all EEG electrodes. Due to equipment malfunction, fNIRS emitters 1, 3, 4, 5, or 10 were not operational during some sessions. In these cases, fNIRS emitter positions were adjusted to preserve as many channels as possible. Figure 1 shows the positions of EEG sensors and fNIRS channels common to all the sessions.

FES electrodes were placed over the extensor muscles of the wrist and fingers of each hand. Before each session, the stimulation current intensity was individually calibrated to the threshold that produced visible wrist extension of 30–90°. Neuromuscular electrical stimulation was delivered using the Omnistim-04 (VNIIIMP-VITA, Moscow, Russia), a commercially available medical stimulator. To enable BCI-driven remote control of stimulation intensity, a custom add-on module was attached to the stimulator: electrical motors within the module rotated the device’s built-in intensity knobs, replicating manual operator adjustments. Mechanical travel limiters within the module enforced a predefined maximum current level, allowing full BCI integration without any modification to the stimulator’s internal circuitry.

The stimulation parameters were as follows: pulse frequency, 70 Hz; bipolar shape; pulse width, 0.35 ms; maximum current amplitude allowed by the device, 100 mA. Individual current amplitude limits for each limb were determined for each subject prior to daily sessions. Amplitudes were set to elicit the movement with minimum reported discomfort.

During each session, participants performed kinesthetic motor imagery of right or left wrist extension, alternating with rest periods. Trial-by-trial instructions indicating the hand to imagine or signaling a rest period were displayed on a monitor. The BCI system decoded motor imagery signals and issued corresponding FES commands to the target hand muscles.

The tasks were cued by changing the color of one of three gray arrows displayed around a circle located at the screen center. Blue color indicated preparation, and green color served as a go signal. Motor imagery trials lasted for 9 s with a 1.5 s preparatory period. Four motor imagery tasks (2 for left wrist and 2 for right wrist) constituted a block. Seven blocks constituted one session, which lasted 595 s (Figure 1c).

EEG data were classified using a filter-bank multiclass common spatial pattern (MCSP) algorithm combined with a shrinkage linear discriminant analysis (sLDA) classifier. The filter bank included Butterworth filters with theta (4–7 Hz), alpha (7–13 Hz), and beta (13–30 Hz) passbands, each filter combined with a 50 Hz notch filter. The highest cutoff frequency, 30 Hz, was more than two times lower than the FES stimulation frequency. Visual inspection of the filtered EEG signal during FES threshold search did not reveal any noticeable artifacts. The fNIRS wavelength data were converted into oxyhemoglobin (HbO) and deoxyhemoglobin (Hb) concentrations. These signals were filtered using a high-pass Chebyshev filter with a 0.01 Hz cutoff frequency and a band-pass Chebyshev filter with a passband depending on the frequency of the rest cue presentation. The band-pass filtered fNIRS data were passed to an sLDA classifier for distinguishing rest and motor imagery; left- and right-hand motor imagery were treated as the same class. If the first NIRS classifier detected motor imagery, the high-pass-filtered NIRS data were then passed to a second NIRS sLDA classifier to determine which wrist movement was being imagined. Unlike single-trial paradigms, resting periods preceding the motor imagery tasks were not used to derive a baseline signal to be subtracted from the motor imagery response; the rest was treated as a separate class. Both EEG and NIRS classifiers used all the available channels of the corresponding modality. For each session, the classifiers were trained from scratch after the first block of instructions and updated after each subsequent block. Despite different classification pipelines, both the EEG and NIRS classifiers solved the three-class discrimination tasks. The classifiers made the decisions independently. Thus, to combine their responses during hybrid EEG–fNIRS classification, the probabilities returned by the classifiers were averaged, and a class with the maximum value was chosen. In order to obtain three probabilities for NIRS responses, the resting state probability was derived from the first NIRS sLDA classifier, and the motor imagery probabilities were derived from the second one and multiplied by the probability of the first classifier to detect motor imagery. Classification outputs were generated every 250 ms based on the latest 1 s data epoch. If the classifier correctly recognized the active state, that is, left- or right-hand motor imagery, the stimulation current amplitude began to increase at a constant rate of 16 mA/s. If classification was incorrect, the amplitude decreased at the same rate. When the instruction changed, the amplitude dropped to zero. During each trial, only the stimulation amplitude of the cued hand was adjusted; stimulation of the other hand was not triggered, regardless of classifier output. No feedback was presented during rest.

### 2.5. fNIRS Classification Simulation and Optimal Channel Set Search

To compare classification accuracy using each modality (EEG or fNIRS) separately, a classification simulation based on fNIRS signals was performed for all the sessions of all the participants. The signal processing and classification parameters were identical to those used in the online classification procedure and are described in the previous subsection.

The simulations were also used to estimate classification accuracy for 4 two-class problems: Rest vs. Left hand motor imagery, Rest vs. Right hand motor imagery, Left hand motor imagery vs. Right hand motor imagery, and Rest vs. motor imagery when Left and Right hand motor imagery were combined into the same class. Simulations were performed for three signal modalities: EEG, NIRS, and EEG + NIRS. The resulting accuracy estimates were compared across modalities using the Friedman test for each of the two-class problems.

To assess whether an alternative fusion approach, rather than probability-level fusion, would noticeably affect BCI performance, we performed additional simulations using combined EEG and fNIRS data. EEG features were extracted in the same way as in the experimental online procedure. fNIRS features comprised HbO and Hb concentration signals filtered using both filters, resulting in four feature vectors, which were concatenated with the EEG feature vectors. The combined feature set was classified using Random Forest (256 trees, unconstrained depth, max. features = $\sqrt{n_{features}}$, no class weights), sLDA, and SVM (RBF kernel, C = 1, kernel scale = $\sqrt{\frac{n_{features}var\left(X\right)}{2}}$, one-vs-one multi-class scheme, no class weights) classifiers. For sLDA and SVM, feature-wise z-scoring was applied to the test-set feature vectors using the mean and standard deviation estimated from the training set.

Both simulated and online classification accuracy was evaluated using macro-averaged recall, hereafter referred to as recall. Because recall can be interpreted as the average probability of correct recognition, it will be reported as a percentage.

Clinical application of a BCI system requires reducing the number of recording channels to shorten preparation time and minimize patient discomfort. For this reason, we assessed the importance of EEG and fNIRS channels for classification in order to select a reduced channel set for upcoming clinical studies. To this end, we applied a binary genetic algorithm (GA) to identify the individually optimal channel sets for each session, and estimated the overall probability of each EEG and fNIRS channel belonging to the best set. The population consisted of binary masks indicating which EEG and fNIRS channels were included in a given configuration. Only those fNIRS channels which were present in all the sessions were considered. Note that the channels were defined by actual emitter and detector positions but not by the optode numbers. The algorithm optimized the mean F1-measure across left wrist and right wrist movement imagery classes, derived from hybrid classification simulations. The exact cost function was

(1) $$1-\frac{1}{2}\left(F_{1}^{LH}+F_{1}^{RH}\right)+\lambda \frac{m}{C}$$

where *m* is the number of selected channels, *C* is the number of all EEG and fNIRS channels, and *λ* is a regularization parameter promoting channel sparsity. To obtain a symmetric montage, channels symmetric with respect to the mid-sagittal plane were added or removed simultaneously. The population size was set to 250, and each run terminated after 60 generations. Based on inspection of channel inclusion probabilities, we selected two final montages for subsequent clinical studies, which differed in the number of EEG channels used. Classification recall and F1-measures for both movement imagery classes were then estimated for these reduced sets using the simulation algorithm.

### 2.6. Participant Questionnaire

Participants’ subjective experiences during BCI–FES sessions and any potential adverse effects were assessed using a purpose-designed questionnaire developed specifically for this study (Appendix A). The instrument comprised four blocks covering sense of agency, sustained attention, physical comfort, and overall impression of the procedure. All items were rated on a bipolar scale from −3 to +3. The agency block captured the perceived sense of movement control, the temporal contingency between motor imagery and muscle contraction, and the subjective clarity of the causal link between imagined and evoked wrist extension. Because agency was assessed separately for each hand across 3 questions, the possible score range for this questionnaire block −18 to 18.

### 2.7. Study Dataset

The dataset is freely available at the open access NeuroImaging Tools & Resources Collaboratory (NITRC) repository [https://doi.org/10.25790/bml0cm.200]. The data are available at the link [https://www.nitrc.org/projects/eeg_nirs_fes (accessed on 31 August 2026)] under the Attribution 4.0 International (CC BY 4.0) license. The dataset is provided in BIDS format. EEG data are stored in EDF format, and fNIRS data are stored in SNIRF format.

To validate the EEG data, we computed average log-transformed ERD/ERS for the C3 and C4 EEG channels. ERD/ERS was estimated for each continuous 9 s motor imagery epoch preceded by a 1.5 s preparation period, using the last 5 s of the preceding rest interval as baseline. To obtain ERD/ERS as a function of time, we first estimated instantaneous band power at each time point in the alpha (8–13 Hz) and beta (13–30 Hz) bands. Band power was computed as the variance of the band-pass filtered signal within a 300 ms time window centered on the time point of interest. Log-transformed ERD/ERS was then calculated as

(2) $$E=\log_{10}\left(\frac{P_{band}\left(t\right)}{P_{base}}\right)$$

where $P_{band}\left(t\right)$ is the instantenious band power at time *t* and $P_{base}$ is baseline band power computed for last 5 s of the rest epoch. The ERD\ERS values were averaged across all epochs within each session, then across all sessions for each subject, and finally across all subjects.

To validate the fNIRS data, we calculated the averaged HbO and HbR concentrations from two symmetrical channels during motor imagery. To reduce the contribution of sessions with high gain and a low signal-to-noise ratio, the HbO and HbR signals from each session were z-scored. The signals were also filtered using a second-order zero-phase Chebyshev filter with a passband of 0.005–0.5 Hz. Each epoch started 9 s before the onset of the active instruction and ended 20 s after it. The 5 s interval preceding the instruction onset was used as the baseline. All epochs from all sessions and all participants were then averaged.

### 2.8. Statistics

Statistical analyses were performed using Statistica 6.0 (StatSoft, USA). Because of the relatively small sample size, nonparametric tests were applied. The Mann–Whitney U test was used to compare quantitative variables between two independent samples, the Wilcoxon signed-rank test was used to compare quantitative variables between two related samples, and Spearman’s rank correlation was used to assess correlations between variables. Quantitative data are reported as median with 25th and 75th percentiles. The level of statistical significance was set at *p* < 0.05. For the ten pairwise comparisons of classification metrics reported in Section 3.5, Bonferroni correction was applied, with a corrected significance threshold of *p* < 0.005.

## 3. Results

### 3.1. Study Subjects

Eighteen healthy volunteers were initially enrolled. Two participants (both female, aged 23 and 28 years) were subsequently excluded due to insufficient individual sensitivity to FES, yielding a final sample of 16 volunteers (11 females, 5 males; median age 23 [21.0; 23.5] years, range 20–28 years). Participants were randomly assigned to one of two age-matched groups (*p* > 0.05). Initially, nine participants were randomized to the hybrid EEG–fNIRS BCI group and seven participants to the EEG-only BCI group. However, in one participant from the hybrid group, the fNIRS system malfunctioned during the first and second training days; therefore, feedback was delivered based on EEG signals only. Because the study was blinded, this participant was not aware of the group assignment at any point, and the malfunction therefore did not represent a mid-study switch in perceived or behaviorally relevant conditions: the FES feedback actually delivered to this participant was driven exclusively by EEG-derived classification from the first training day onward, identical in nature to the feedback delivered to participants nominally randomized to the EEG-only group. This participant (Subject 05) was therefore reassigned to the EEG-only BCI group prior to the third training day by a researcher not involved in conducting the experimental sessions, correcting the group label to reflect the control modality functionally in effect throughout, rather than the nominal randomization assignment. As a result, each group comprised eight participants. The hybrid EEG–fNIRS BCI group comprised seven females and one male (median age 23.0 [21.0; 23.5] years); the EEG-only BCI group comprised four females and four males (median age 23.0 [20.5; 23.5] years).

### 3.2. Dataset Validation

There were 80 sessions across 16 participants. fNIRS data were missing in 11 sessions across four participants: the last 2 sessions of Subject 02 (EEG–fNIRS BCI group), resulting in exclusion of the final training day from the accuracy and agency analyses; the last 2 sessions of Subject 03 (EEG-only BCI group); the last 4 sessions of Subject 04 (EEG-only BCI group); and the first 3 sessions of Subject 05, who was subsequently reassigned to the EEG-only BCI group prior to the third training day.

**Figure 2:**
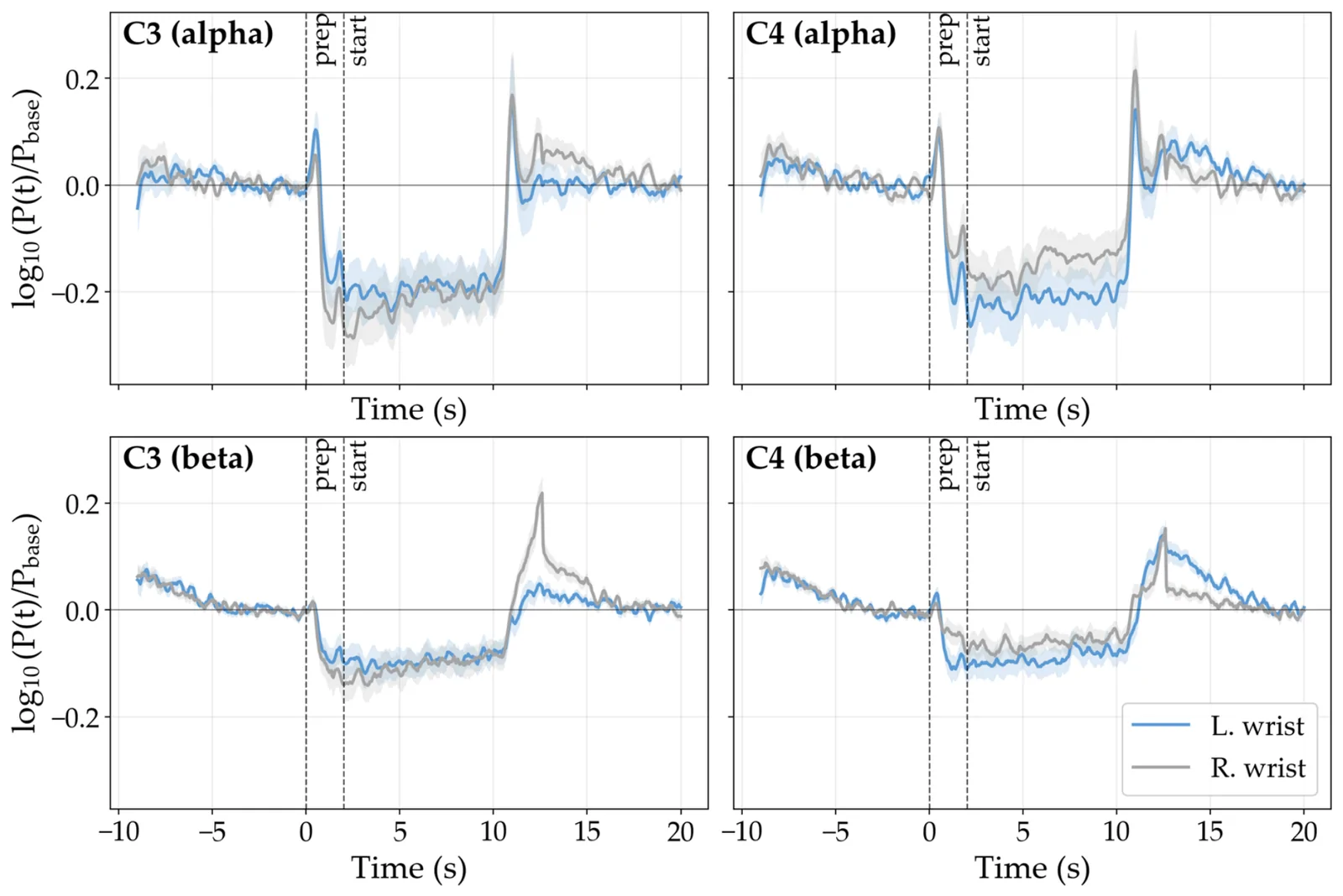
Average log-transformed EEG ERD\ERS plots for C3 and C4 channels obtained for left and right wrist movement imagery and two frequency bands. The horizon line indicates zero level (no synchronization or de-synchronization) and areas indicate standard error.

The average log-transformed ERD\ERS plots are shown in Figure 2. There is evident asymmetric average desynchronization lasting throughout the motor imagery epochs, followed by synchronization rebound.

**Figure 3:**
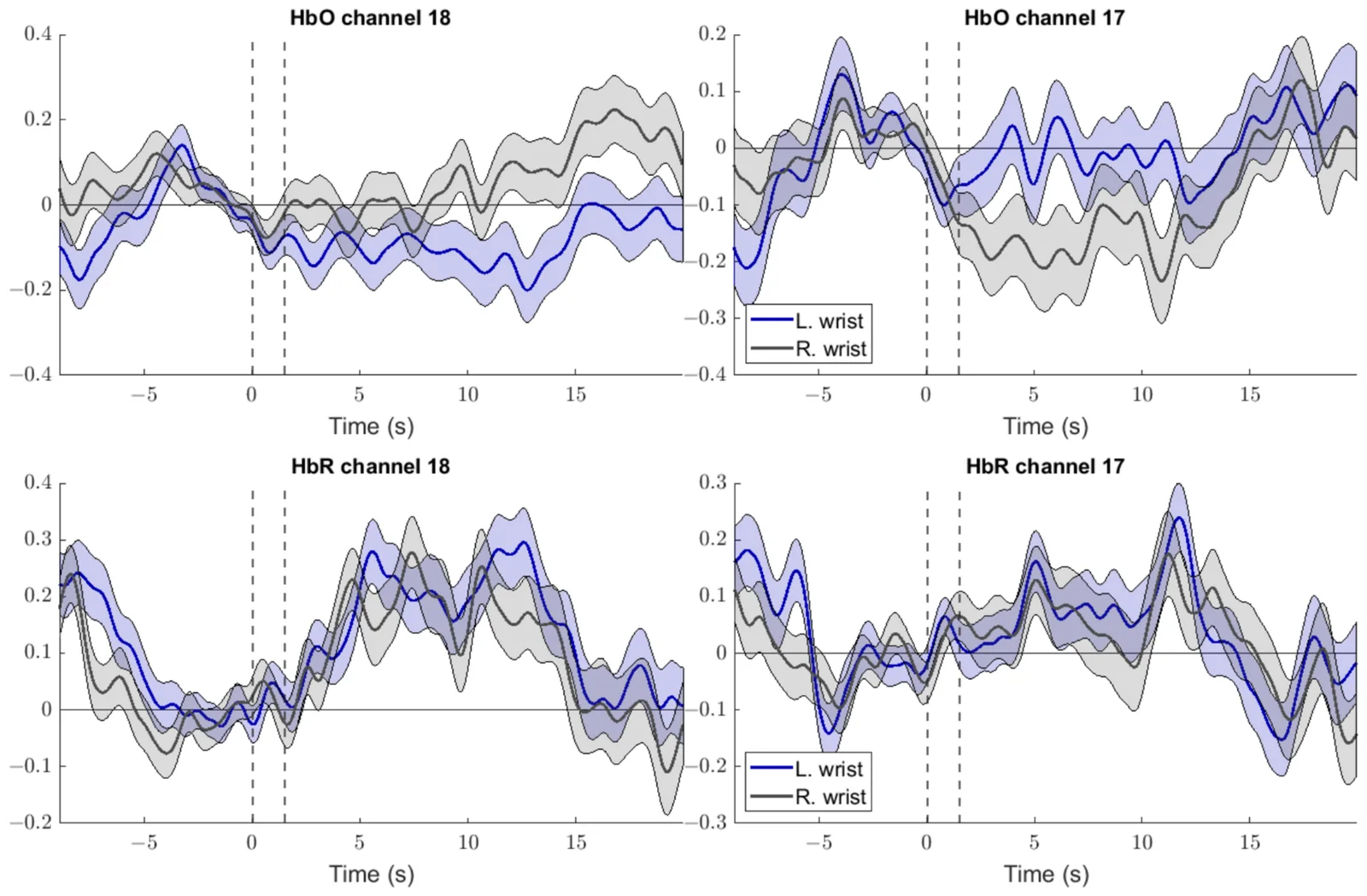
Average HbO and HbR responses to motor imagery tasks. Channel numbers correspond to those in Figure 1b. The horizon line indicates zero level (no synchronization or de-synchronization) and areas indicate standard error.

Asymmetric changes in HbO concentration are observed in symmetrical channels located in opposite hemispheres (Figure 3). Zero time corresponds to the onset of the motor imagery preparation phase. The motor imagery task onset is indicated by the second vertical line at 1.5 s.

### 3.3. Hybrid EEG–fNIRS BCI and EEG BCI Performance

#### 3.3.1. Classification Accuracy

**Figure 4:**
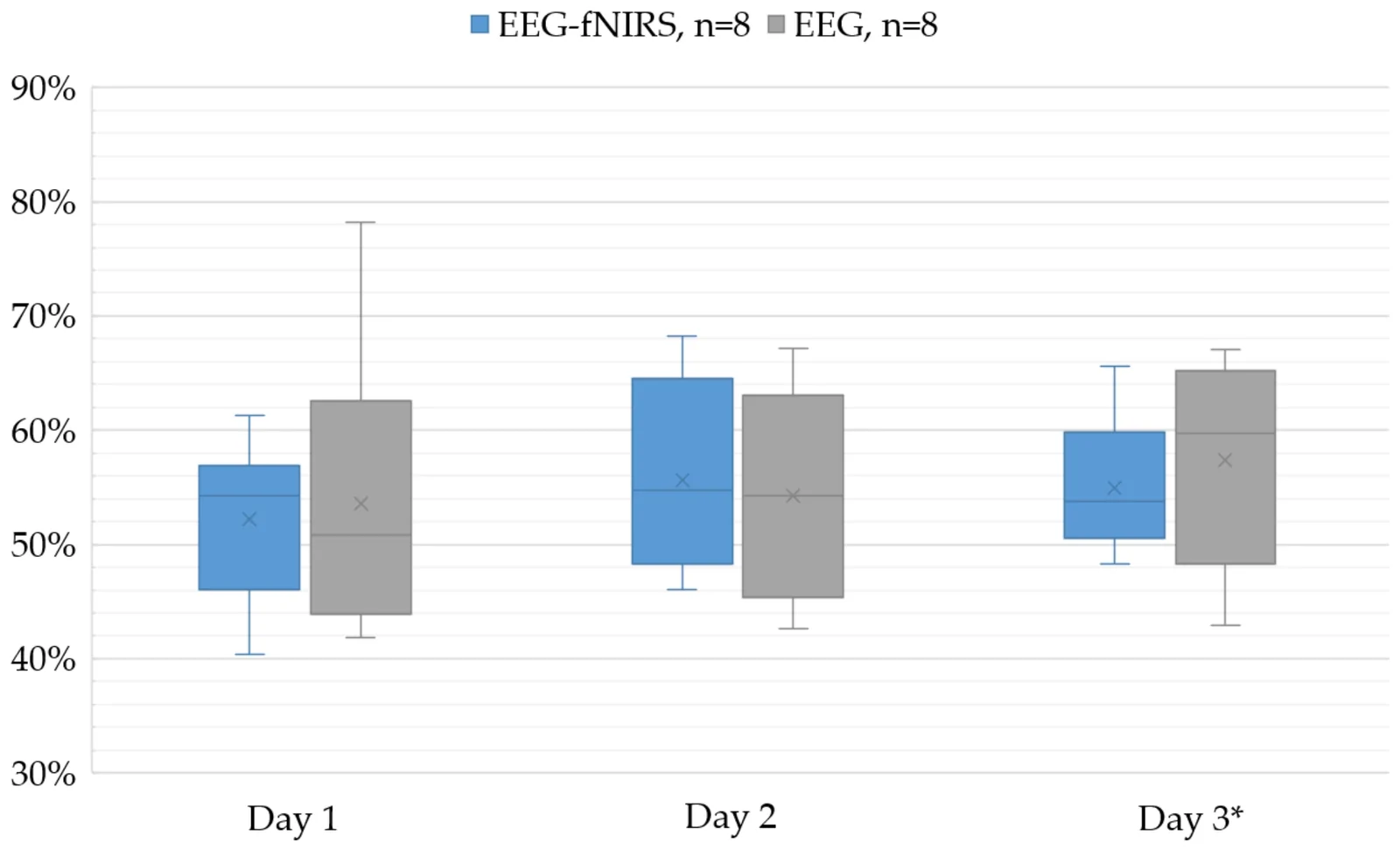
Recall on different training days. Box boundaries represent the 25th and 75th percentiles; the horizontal line inside the box indicates the median; the cross indicates the mean; whiskers represent the minimum and maximum values. * The data for one participant in the EEG–fNIRS BCI group were excluded on day 3, because the fNIRS system was not functioning that day.

Online classification recall exceeded chance level (33.3% for a three-class paradigm) in both groups and did not differ significantly between conditions. Mean recall averaged across the three training days was 53.5 [51.0; 59.4]% for the hybrid EEG–fNIRS BCI and 57.3 [45.6; 61.6]% for the EEG-only BCI (*p* = 1.00). The maximal achieved recall was 56.4 [55.0; 66.2]% and 64.1 [51.4;68.5]%, respectively (*p* = 1.00). Recall did not differ significantly between groups on any of the three training days (*p* > 0.05, see Figure 4).

**Figure 5:**
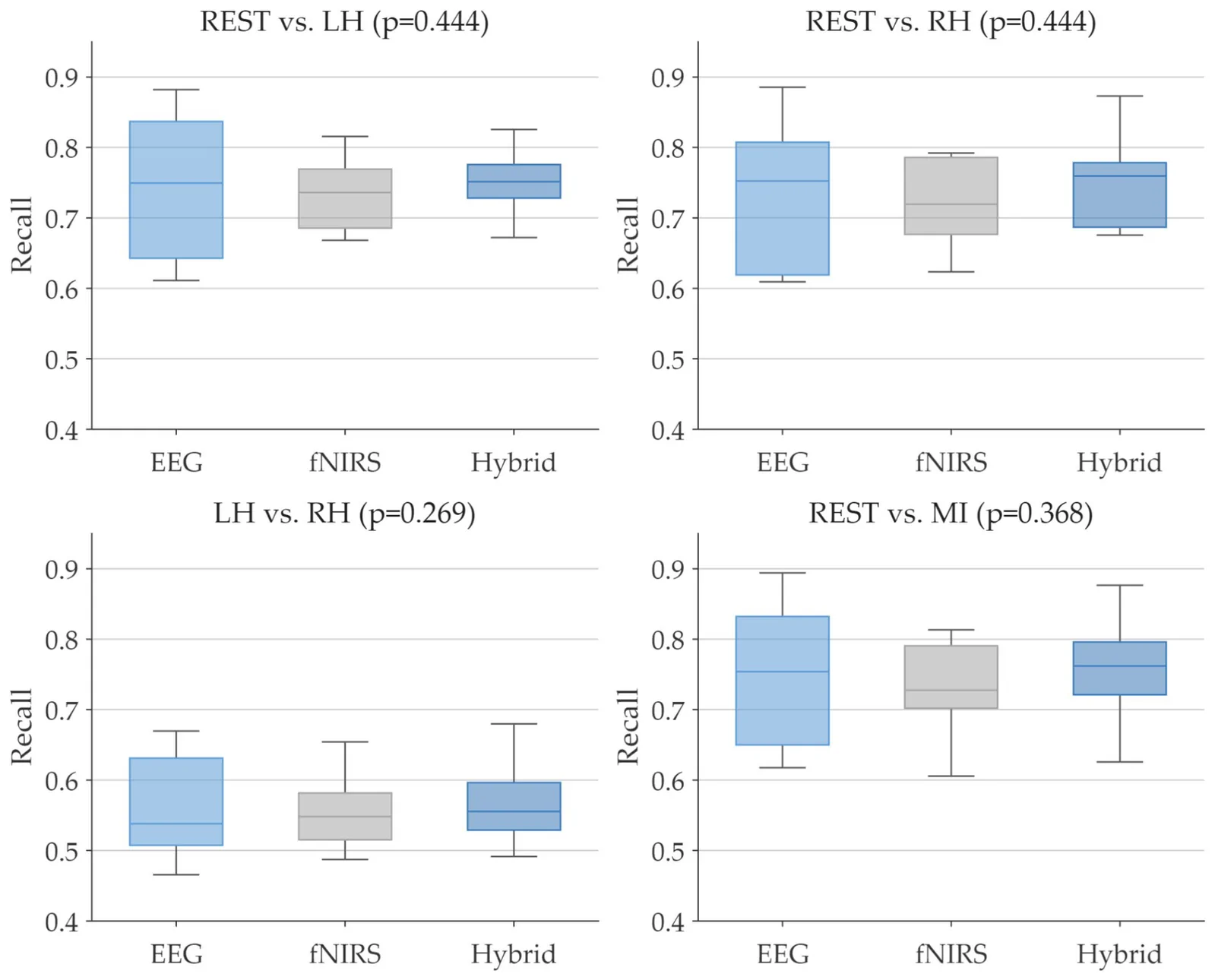
Pairwise classification accuracy for different signal modalities.

Figure 5 shows average recall values obtained with pairwise classification simulations for different signal modalities. There were no significant differences between the modalities for any of the class pairs (Friedman *p* > 0.27).

The simulations using different feature fusing strategies resulted in average recall values of 59.2 [53.7; 66.4]% for Random Forest, 56.3 [54.3; 64.7] for sLDA, and 59.7 [55.5; 66.9]% for SVM. The difference was significant (Friedman *p* = 0.003): the original hybrid classifier slightly outperformed the Random Forest and sLDA (Wilcoxon *p* = 0.02 and *p* = 0.035 after Bonferroni correction). The performance of the hybrid and the SVM classifiers differed insignificantly (corrected Wilcoxon *p* = 0.128).

#### 3.3.2. Agency

**Figure 6:**
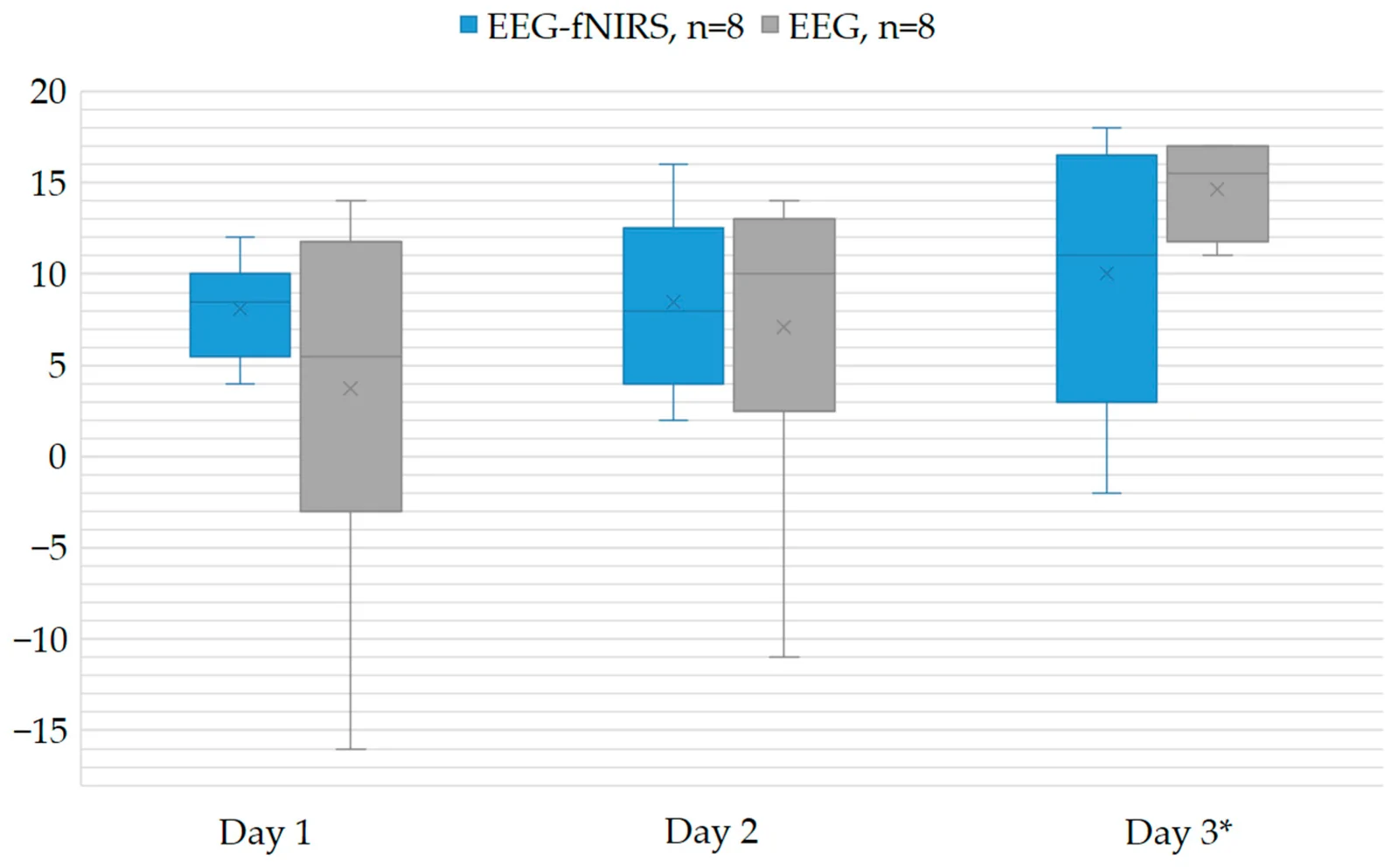
Sense of agency over hand movements on individual days. Box boundaries represent the 25th and 75th percentiles; the horizontal line inside the box indicates the median; the cross indicates the mean; whiskers represent the minimum and maximum values. * The data for one participant in the EEG–fNIRS BCI group were excluded on day 3, because the fNIRS system was not functioning that day.

Agency, defined as the subjective sense of control over movement, did not differ significantly between the groups on any of the three study days (*p* > 0.05; see Figure 6). The median agency score averaged over the three days was 9.0 [4.5; 11.1] in the EEG–fNIRS group and 7.7 [3.7; 12.0] in the EEG-only group (*p* = 0.878), corresponding to 75.0% and 71.4% of the maximum possible value on the −18 to +18 scale, respectively. Agency scores did not correlate with classification accuracy on any training day (*p* > 0.05).

#### 3.3.3. Stimulation

The average individual maximum current amplitudes defined before the sessions were 28.7 [24.7; 33.9] mA for the left wrist and 26.7 [24.2; 28.9] mA for the right wrist.

The median duration of muscle electrical stimulation for both hands per session was 132.59 [110.39; 140.10] sec in the EEG–fNIRS BCI group and 149.10 [122.31; 162.43] sec in the EEG-only BCI group (*p* = 0.234).

Given the clinical relevance of assessing the stimulation dose delivered to a single paretic limb in post-stroke rehabilitation, we additionally calculated this parameter separately for one hand (the right hand in this sample) across all subjects and the three training days. The median value was 299.23 [263.16; 338.32] sec, or 4.99 [3.94; 5.64] min.

#### 3.3.4. Sensitivity Analysis for Group Reassignment

Because Subject 05 was reassigned from the hybrid EEG–fNIRS group to the EEG-only group (Section 3.1), we performed a sensitivity analysis to verify that this reassignment did not bias the reported between-group comparisons. This analysis excluded Subject 05 entirely from the EEG-only group, reducing its sample size from 8 to 7 participants.

Exclusion of this participant did not meaningfully change the group’s median performance metrics. Average recall was 57.9% [44.5; 61.9] compared with 57.3% [45.6; 61.6] in the original analysis, and maximum recall was 65.7% [50.9; 69.8] compared with 64.1% [51.4; 68.5]. Between-group comparisons of recall remained non-significant across all measures (*p* > 0.05), consistent with the original analysis including all 16 participants. Average ERD/ERS values likewise showed no meaningful change following exclusion of this participant.

Given that excluding Subject 05 did not alter any of the study’s conclusions, we retained this participant and their data in the final dataset and primary analysis reported throughout this manuscript.

### 3.4. Attention and Fatigue

Across the full sample, the median averaged subjective vigilance rating over the 3 days was 3.0 [1.8; 4.2] points, corresponding to 67% of the maximum possible value on a scale from −6 to +6. Seven of the sixteen participants reported mild fatigue and reduced concentration by the middle or end of a session, predominantly on the first day. The most frequently cited sources of distraction were external sounds (n = 2), somatic discomfort (n = 2), and pre-existing fatigue (n = 2).

Subjective attention and fatigue scores did not differ between the groups on any of the three days (*p* > 0.05 for all comparisons), and complaints of reduced concentration or discomfort occurred at comparable rates in both groups.

### 3.5. Safety and Tolerability for Subjects

Of the 18 screened volunteers, 2 were excluded due to a low current sensitivity threshold.

Across the full sample, the median of subjective comfort ratings over 3 days was 4.3 [3.7; 5.3] points, corresponding to 86% of the maximum possible value on a scale from −6 to +6. Five participants reported discomfort attributable to constrained body position (arm numbness, back pain), and one participant reported discomfort related to room temperature.

### 3.6. Simulation of Feedback Based Separately on fNIRS and on EEG

Based on an analysis simulating the use of each modality in isolation, control accuracy with feedback derived exclusively from fNIRS versus exclusively from EEG did not differ significantly. The within-subject average recall was 53.08 [49.21; 57.55]% for fNIRS and 56.60 [47.55; 61.62]% for EEG (*p* = 0.379), while the within-subject maximum recall values were 59.40 [57.35; 63.99]% and 62.84 [52.41; 68.50]%, respectively (*p* = 0.836).

### 3.7. Determining the Optimal Number of Electrodes

**Figure 7:**
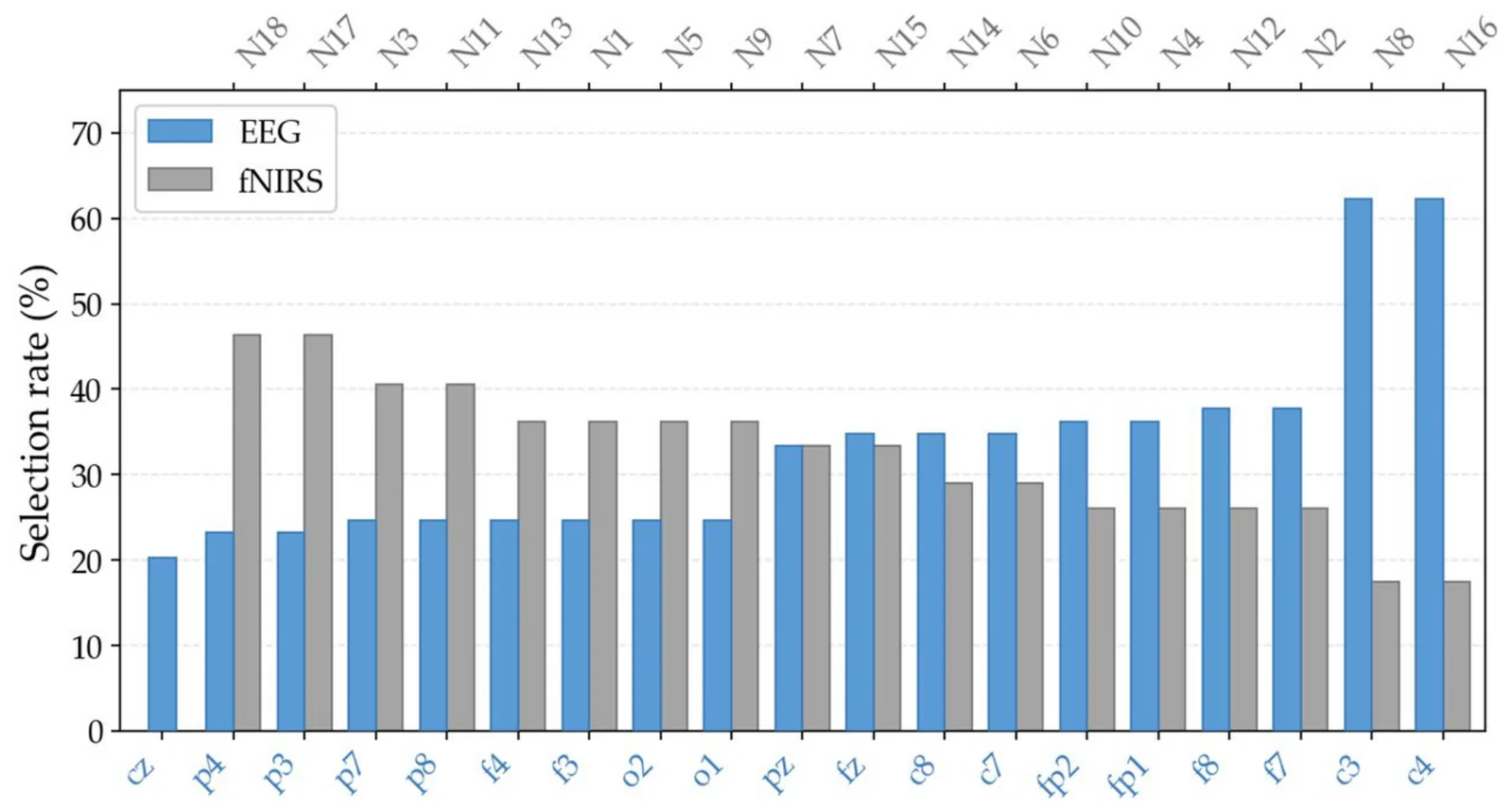
Rates of EEG and fNIRS channel inclusion in the best set according to the GA search. fNIRS channel numbers correspond to numbers in Figure 1c.

The GA optimal channel search was performed for 80 sessions, of which 69 sessions had both EEG and fNIRS data available. Eleven sessions were missing the fNIRS data. On average, the GA selected 6.7 ± 3 EEG channels and 5.7 ± 2.7 fNIRS channels per session. The channel selection rates are presented in Figure 7. As expected, the best EEG channel sets were more likely to include C3 and C4 channels. For the fNIRS channels, no clear selection threshold was evident; therefore, we selected the eight channels that most frequently appeared among the best-performing channel sets.

The average recall obtained in simulations using the manually selected channel set was 58.4 [50.3; 61.4]% (*p* = 0.156), which did not significantly differ from the online recall of 56.5 [50.5; 63.5]%. The average F1-measure for motor imagery classes was also close to the online value: 0.465 [0.375; 0.498] vs. 0.44 [0.38; 0.55] (*p* = 0.255). Using the best-performing channel set significantly improved classification compared to the online performance. Recall increased to 63.2 [57.8; 66.2]% (*p* = 0.00043), and the average F1-measure for motor imagery classes increased to 0.503 [0.464; 0.572] (*p* = 0.00053). 

Analysis of the simulation results obtained separately for EEG and fNIRS classifiers before voting showed that using only two EEG channels yielded 50.3 [46.4; 57.2]% recall and 0.422 [0.377, 0.494] average F1-measure for the motor imagery classes. The performance indices did not differ significantly from those obtained in the hybrid classification simulation (*p* > 0.3 for both recall and F1-measure). Using only the selected fNIRS channels resulted in significantly lower indices: the recall decreased to 49.3 [43.5; 53.4]% (*p* = 0.000031) and the average F1-measure decreased to 0.378 [0.319; 0.401]% (*p* = 0.000092). 

However, using only EEG or only fNIRS channels from the best sets resulted in lower classification indices compared to the best EEG–fNIRS classification. For EEG, the indices became 58.7 [50.8; 61.8]% (*p* = 0.000031) and 0.479 [0.408; 0.527] (0.000031), and for fNIRS, the indices became 58.1 [52.2; 63.1]% (*p* = 0.0016) and 0.474 [0.424; 0.523] (*p* = 0.007629, which did not survive Bonferroni correction).

## 4. Discussion

In a blinded randomized controlled study involving healthy volunteers, we demonstrated comparable performance of two types of real-time, three-class BCIs controlling FES: one based on combined EEG and fNIRS recording, and one based on EEG recording alone. These interfaces did not differ in recall, subjective sense of agency, or FES activation time. To the best of our knowledge, this is the first study to compare a hybrid and a unimodal BCI for online FES control in a three-class paradigm. Its result challenges the view that hybrid recording of electrical and hemodynamic signals necessarily confers an advantage over a unimodal system [11].

Comparisons of simulated classification metrics for EEG-only and fNIRS-only approaches suggest that, on average, both methods can be used in clinical BCIs with comparable accuracy. One possible explanation for this finding is that both the EEG and fNIRS classifiers extract features reflecting coupled neurophysiological phenomena underlying motor imagery. Specifically, CSP spatial filters maximize the ratio of band-power between classes, thereby capturing EEG rhythmic components associated with event-related synchronization and desynchronization. These EEG changes are accompanied by local changes in HbO and HbR concentrations detectable by fNIRS. For EEG feature extraction, we used the multi-class filter bank CSP, as the CSP algorithm and its variants remain among the most widely adopted feature extraction methods in EEG-based BCIs and monitoring systems [25,26], including systems that incorporate ANNs and AI-based approaches [27]. We used the fNIRS classification algorithm previously validated in our earlier clinical fNIRS-BCI study [10], where it demonstrated superior performance over EEG-BCI in post-stroke patients, as was shown, however, by retrospective analysis only.

It should be emphasized, however, that this conclusion pertains specifically to the present sample size and relatively simple fusion approach implemented here. However, the simulations utilizing combined EEG and NIRS feature vectors indicate that the approach provides the same or slightly better performance in case conventional machine learning techniques are used for classification. Still, it remains possible that more advanced approaches, such as dedicated ANN architectures that jointly process EEG and fNIRS signals rather than combining independent classical machine learning pipelines, could reveal a hybrid advantage [28]. While the statistical analysis supports this finding within the current experimental setting, the limited sample size and the absence of comparisons with more advanced multimodal fusion methods restrict its generalizability. Because the main goal of this study was to evaluate the practical performance of the hybrid BCI, including its online performance, a systematic comparison of multiple classifiers was beyond the scope of the work. Nevertheless, we provide the obtained data in the hope that they will be useful to other teams engaged in designing rehabilitation BCIs.

In the majority of studies employing hybrid EEG–fNIRS BCI recordings in healthy subjects, signal classification was performed offline, using either artificial neural network-based approaches [13,14,16,17,18,19] or simpler feature extraction and classification methods such as combining CSP with SVM, or LDA [12,15,20]. In the few studies where online classification was implemented, a binary classifier distinguishing between two states was typically used [21,22,23,24,29,30]. Notably, only one of these online studies performed classification using a genuinely hybrid classifier that combined both EEG and fNIRS-derived features into a single feature vector: two fNIRS-derived and two EEG-derived features were combined into a four-dimensional feature vector and classified using LDA [22]. Taken together, these observations indicate that direct evidence on the practical performance of hybrid EEG–fNIRS BCIs under real-time, closed-loop conditions remains scarce, and that existing online implementations have been largely restricted to binary classification tasks. To our knowledge, the present study is the first to evaluate a hybrid EEG–fNIRS BCI against an EEG-only BCI in an online, three-class, closed-loop paradigm directly driving FES feedback. This distinction may be relevant, as offline decoding accuracy is known to overestimate real-world performance, and binary classification may not capture the added complexity of discriminating between multiple motor imagery states, as required for practical BCI–FES applications in motor rehabilitation.

The optimal channel configuration search showed that selecting two EEG channels, C3 and C4, may be sufficient for the chosen classifier to work with sufficient accuracy. In contrast, no fNIRS channels were consistently identified as the most informative across the participants. Reducing the fNIRS channel set resulted in a decrease in group-averaged classification metrics, suggesting that either an extended or individually optimized channel configuration is required. Several factors may explain this finding. First, fNIRS can be substantially contaminated by superficial scalp vasculature [31], which is unrelated to neural activity. Second, fNIRS channels detect focal concentration changes in cortical areas directly beneath each source-detector pair. Together, these factors highlight the need for careful, individually tailored sensor placement, ideally guided by structural and functional MRI data [32], which were unavailable for our participants. It should be noted that individual MRI and fMRI data are rarely available even for hospitalized patients undergoing rehabilitation. Although standardized head models and publicly available statistical activation maps could be used as surrogates, this approach would still require head digitization before each session, likely increasing preparation time and patient burden. Finally, the concurrent use of multi-channel EEG and fNIRS caps imposes additional constraints on fNIRS optode placement, limiting the freedom to position optodes at individually optimized scalp locations.

Although the simulation analysis demonstrated comparable classification accuracy for unimodal fNIRS-based and EEG-based BCIs for the three- and two-class cases, future studies should directly compare these configurations in the context of FES-driven motor rehabilitation, with particular attention to the sense of agency. Rapid sensory feedback is especially important in FES applications, and the slow, delayed hemodynamic response inherent to fNIRS may negatively impact this subjective outcome measure. Related to this delay, the extent to which prolonged post-task hemodynamic changes from preceding trials may influence classification of the subsequent trials in short-interval, continuous motor imagery paradigms such as ours also warrants further careful investigation.

The majority of the BCI systems tested and applied in clinics follow the approach that requires stimulation of only the affected hand or otherwise reinforcing its motor imagery. Two-class discrimination is sufficient for the task, and it can be achieved using as simple a classification strategy as determining a sensorimotor rhythm threshold for two-class discrimination between resting state and motor imagery [33,34,35,36]. A recent randomized controlled trial with most participants to date [37] used a CSP and SVM combination for the two-class task, while several earlier works utilized the FBMCSP approach [38,39]. Notably, commercially available RecoveriX by g.Tec, one of the leading non-invasive BCI companies, still uses the CSP + LDA approach. Technically, a two-class approach yields higher accuracy metrics, as suggested both by the results reported in the aforementioned papers and the two-class simulations. We, however, made a different design choice for our real-time system to enable three-class classification, which more effectively supports the discrimination of motor imagery states. A rest-versus-motor-imagery paradigm is inherently less demanding in terms of specificity, as classifiers may capture non-specific correlates of attention or mental effort rather than true lateralized motor cortex activation, unless feature selection explicitly constrains the relevant spatial and frequency components. In addition, bilateral training after stroke is thought to enhance both intra-hemispheric and interhemispheric connectivity within the sensorimotor network through activation of the ipsilesional primary motor cortex, supplementary motor area, and primary somatosensory cortex [40].

In the present study, we also assessed a number of subjective parameters in participants to inform the development of clinical protocols. The sense of agency was examined not to analyze its relationship with BCI control accuracy or neurophysiological measures, but to evaluate the participants’ subjective perception of the procedure. We suggest that the operator’s feeling that “my thought triggers my movement” may substantially influence their acceptance of the system and engagement with the control process. Although agency has a complex psychophysiological nature and may depend on the context of the BCI control procedure [41], it can also be considered a useful metric of BCI performance. Given the aims of the present study, agency was assessed using a simplified questionnaire developed with reference to established embodiment research methodology [42]. This subjective metric did not differ between the hybrid and unimodal BCI groups.

The total effective duration of electrical stimulation per session did not differ significantly between the groups. The estimated stimulation time for a single hand was approximately 5 min across 3 training days. Given that each session in the present protocol lasted approximately 10 min and the full training course consisted of 5 sessions across three days, future clinical protocols should increase both the total number of training sessions (up to 10–20, as reported in previous clinical studies [43,44,45,46]) and the duration of each individual session. However, given that fatigue is a common and clinically significant concern in post-stroke patients, individual training sessions should generally not exceed 30–40 min, and future clinical protocols should incorporate real-time fatigue monitoring along with adaptive session pacing.

The present study has several limitations. The study was conducted on a relatively small sample. Some fNIRS data were lost due to a device malfunction on several days; in the EEG–fNIRS group, one participant had a non-functional fNIRS recording during the final training day, and this session was therefore excluded from the classifier accuracy analysis. We implemented a relatively simple fusion approach, which may have limited our ability to fully exploit the complementary strengths of EEG and fNIRS. One participant’s group assignment deviated from strict randomization due to equipment malfunction (a sensitivity analysis excluding this participant did not alter the study conclusions). Finally, it is important to emphasize that the recommendations for clinical protocols outlined above, as well as the study’s overall conclusions regarding modality choice, were derived from data obtained in healthy volunteers who were, on average, relatively young (median age 23 years, range 20–28 years). Although the inclusion criteria allowed participation of adults up to 65 years of age, the recruited sample was not representative of this broader age range, nor of the typically older post-stroke population. Both EEG and fNIRS signals can be affected by age-related changes or altered by brain changes associated with cerebrovascular disease. However, based on our team’s prior experience applying three-class BCI in clinical populations, including older adults and post-stroke patients, we have not observed systematic differences in real-time BCI control accuracy attributable to age per se: in our earlier clinical fNIRS-BCI [10] and EEG-BCI [47] studies, post-stroke patients achieved classification accuracy comparable to that reported here in younger healthy volunteers.

In principle, stroke-related neurovascular uncoupling could further compromise the specificity of fNIRS hemodynamic signals in some patients. However, in our own comparative clinical work directly contrasting real-time three-class NIRS-BCI and real-time three-class EEG-BCI in post-stroke patients undergoing upper-limb motor imagery training, NIRS-BCI achieved significantly higher control accuracy than EEG-BCI (median control accuracy 46.4% vs. 40.0%, *p* = 0.004; median maximum accuracy 66.2% vs. 50.6%, *p* = 0.006) [10]. In a separate cohort of stroke patients (median age 58 years) using real-time two-class fNIRS-BCI for gait rehabilitation, median maximum classification accuracy reached 75.6%, with no correlation between age and control accuracy [48]. These findings do not support the expectation that fNIRS-based BCI control is systematically impaired in stroke populations. Given that (i) the hybrid EEG–fNIRS BCI provided no measurable advantage over EEG-only BCI in the present study, (ii) simulation analysis revealed comparable classification accuracy for unimodal EEG-only and fNIRS-only classifiers, and (iii) our prior clinical data suggest fNIRS-BCI may achieve even higher control accuracy than EEG-BCI in stroke patients specifically, we recommend that unimodal fNIRS–BCI–FES, rather than the hybrid configuration evaluated here, be prioritized for direct clinical validation as the next research step, taking into account both the practical advantages of fNIRS for clinical use [8,9] and the meta-analytically demonstrated superiority of BCI–FES over other feedback modalities for post-stroke motor recovery [5,6,7].

The BCI–FES dataset recorded for both groups has been made publicly available. We note that this release may represent a useful contribution in its own right, complementing the specific findings of this study. To our knowledge, this is among the first openly available datasets combining simultaneous EEG and fNIRS recordings acquired during real-time, three-class, closed-loop BCI–FES control, rather than offline or simulated paradigms, although the relatively small sample size and incomplete data for some participants should be taken into account when using this resource. Given the current scarcity of online multimodal BCI datasets, we hope that it may support the development and benchmarking of classification and fusion algorithms that were beyond the scope of the present study. It may also be valuable for further research into the neural correlates of motor imagery in the context of post-stroke rehabilitation.

## 5. Conclusions

The practical goal of the present study was to provide a rationale for modifying the BCI and stimulation protocol for subsequent clinical trials. To this end, a number of objective and subjective outcome parameters were assessed. Within the scope of the present study, using a real-time, closed-loop, three-class classification paradigm, the hybrid EEG–fNIRS brain–computer interface provided no advantages over the EEG-only brain–computer interface. Furthermore, unimodal EEG and fNIRS BCIs showed comparable classification accuracy in the feedback simulation yet exhibited distinct profiles across practically relevant characteristics. This suggests that the choice between EEG and fNIRS modalities may need to be made on an individual basis. Future studies should therefore directly compare subjective control outcomes between EEG-based and fNIRS-based BCI–FES configurations.

In addition to these methodological findings, we note that the public release of the complete online EEG-only and hybrid EEG–fNIRS BCI-FES dataset represents a further contribution of this work, offering the research community a resource for developing and benchmarking multimodal decoding algorithms under closed-loop conditions.

## Data Availability

The original data presented in the study are openly available in NITRC at https://www.nitrc.org/projects/eeg_nirs_fes (accessed on 31 August 2026) under the Attribution 4.0 International (CC BY 4.0) license.

## Abbreviations

The following abbreviations are used in this manuscript:

ANN	Artificial neural network
BCI	Brain–computer interface
BIDS	Brain imaging data structure
CSP	Common spatial patterns
EDF	European data format
EEG	Electroencephalography
ERD	Event-related desynchronization
ERS	Event-related synchronization
FES	Functional electrical stimulation
fNIRS	Functional near infrared spectroscopy
GA	Genetic algorithm
HbO	Oxygenated hemoglobin
HbR	Deoxygenated hemoglobin
MCSP	Multiclass common spatial pattern
sLDA	shrinkage linear discriminant analysis
SNIRF	Shared near-infrared spectroscopy format
SVM	Support Vector Machine

## Appendix A

Questionnaire of Subjective Experience of Brain–Computer Interface Control with Functional Electrical Stimulation of hand muscles. Questions are numbered sequentially across all blocks.

**Table A1:** Sense of agency.

Statement	Left Hand	Right Hand
1. I had a sense of controlling the movements that occurred in my hand during the session		
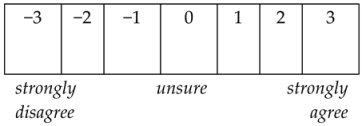
2. During the second half of the session, stimulation was triggered almost immediately after I began imagining the movement		
2a. If you perceived a delay, please estimate the time between the onset of motor imagery and stimulation:	(1) less than 1 s; (2) 1–3 s; (3) 3–5 s; (4) more than 5 s; (5) unsure	(1) less than 1 s; (2) 1–3 s; (3) 3–5 s; (4) more than 5 s; (5) unsure
3. I perceived a clear link between my motor imagery and the moment the stimulation was triggered		

**Table A2:** Attention and fatigue.

Statement	Rating
4. It was easy for me to concentrate and maintain concentration throughout the entire experiment	
5. The experiment did not fatigue me; I feel refreshed after its completion	
At approximately what stage of the procedure did it become difficult to maintain concentration, or when did fatigue occur, if applicable?	

**Table A3:** Physical comfort.

Statement	Rating
6. Electrical muscle stimulation did not cause any unpleasant sensations	
7. During the procedure, my body, arms, and head were in a comfortable position; nothing caused discomfort	
Please describe what caused discomfort, if any:	

**Table A4:** Emotional response.

Statement	Rating
8. I experienced positive emotions during the experiment, and the process was interesting to me	
9. The procedure left me with negative impressions	
